# No effects of oral vitamin D supplementation on non-alcoholic fatty liver disease in patients with type 2 diabetes: a randomized, double-blind, placebo-controlled trial

**DOI:** 10.1186/s12916-016-0638-y

**Published:** 2016-06-29

**Authors:** Ilaria Barchetta, Maria Del Ben, Francesco Angelico, Michele Di Martino, Antonio Fraioli, Giuseppe La Torre, Rosella Saulle, Ludovica Perri, Sergio Morini, Claudio Tiberti, Laura Bertoccini, Flavia Agata Cimini, Francesca Panimolle, Carlo Catalano, Marco Giorgio Baroni, Maria Gisella Cavallo

**Affiliations:** Department of Internal Medicine and Medical Specialties, Sapienza University of Rome, Viale del Policlinico 155, 00161 Rome, Italy; Department of Radiological Sciences, Oncology and Pathology, Sapienza University of Rome, Rome, Italy; Department of Public Health and Infectious Diseases, Sapienza University of Rome, Rome, Italy; Microscopic and Ultrastructural Anatomy (CIR), University Campus Bio-Medico, Rome, Italy; Endocrinology and Diabetes, Department of Experimental Medicine, Sapienza University of Rome, Rome, Italy; Department of Medical Sciences, Endocrinology and Diabetes, University of Cagliari, Cagliari, Italy

**Keywords:** Fatty liver, NAFLD, Vitamin D supplementation, Type 2 diabetes

## Abstract

**Background:**

Non-alcoholic fatty liver disease (NAFLD) is the most common hepatic disorder worldwide, reaching prevalence up to 90 % in obese patients with type 2 diabetes (T2D), and representing an independent risk factor for cardiovascular mortality. Furthermore, the coexistence of T2D and NAFLD leads to higher incidence of diabetes’ complications and additive detrimental liver outcomes. The existence of a close association between NAFLD and hypovitaminosis D, along with the anti-inflammatory and insulin-sensitizing properties of vitamin D, have been largely described, but vitamin D effects on hepatic fat content have never been tested in a randomized controlled trial. We assessed the efficacy and safety of 24-week oral high-dose vitamin D supplementation in T2D patients with NAFLD.

**Methods:**

This randomized, double-blind, placebo-controlled trial was carried out at the Diabetes Centre of Sapienza University, Rome, Italy, to assess oral treatment with cholecalciferol (2000 IU/day) or placebo in T2D patients with NAFLD. The primary endpoint was reduction of hepatic fat fraction (HFF) measured by magnetic resonance; as hepatic outcomes, we also investigated changes in serum transaminases, CK18-M30, N-terminal Procollagen III Propeptide (P3NP) levels, and Fatty Liver Index (FLI). Secondary endpoints were improvement in metabolic (fasting glycaemia, HbA1c, lipids, HOMA-IR, HOMA-β, ADIPO-IR, body fat distribution) and cardiovascular (ankle-brachial index, intima-media thickness, flow-mediated dilatation) parameters from baseline to end of treatment.

**Results:**

Sixty-five patients were randomized, 26 (cholecalciferol) and 29 (placebo) subjects completed the study. 25(OH) vitamin D significantly increased in the active treated group (48.15 ± 23.7 to 89.80 ± 23.6 nmol/L, *P* < 0.001); however, no group differences were found in HFF, transaminases, CK18-M30, P3NP levels or FLI after 24 weeks. Vitamin D neither changed the metabolic profile nor the cardiovascular parameters.

**Conclusions:**

Oral high-dose vitamin D supplementation over 24 weeks did not improve hepatic steatosis or metabolic/cardiovascular parameters in T2D patients with NAFLD. Studies with a longer intervention period are warranted for exploring the effect of long time exposure to vitamin D.

**Trial registration:**

This trial was approved on July 2011 by the Ethics Committee of Policlinico Umberto I, Sapienza University of Rome, Italy, and registered at www.clinicaltrialsregister.eu number 2011-003010-17.

**Electronic supplementary material:**

The online version of this article (doi:10.1186/s12916-016-0638-y) contains supplementary material, which is available to authorized users.

## Background

Non-alcoholic fatty liver disease (NAFLD) is the most common cause of liver disease worldwide [1], with an estimated prevalence of 20 % in the general population and up to 90 % in obese patients affected by type 2 diabetes (T2D) [2, 3]. Diabetes itself, in turn, is capable of accelerating the evolution from NAFLD to non-alcoholic steatohepatitis (NASH), severe fibrosis, cirrhosis and hepatocarcinoma, increasing the liver-related mortality risk [4–7]. Conversely, the presence of NAFLD in T2D patients is associated with worse metabolic profile, greater insulin resistance and higher rate of diabetes’ micro- and macro-vascular complications [8–12]. Indeed, NAFLD is now considered an independent risk factor for cardiovascular mortality [13].

Although several nutraceutical and pharmacological interventions for NAFLD have been proposed, none has shown significant results in an adequate experimental setting, so that NAFLD therapy still remains an open issue [14]. At present, there is no medically approved treatment for NAFLD and a wide variety of nutraceuticals with several modes of action are currently under clinical evaluation.

Contextually to the large evidence on a relationship between low vitamin D levels and metabolic diseases [15–17], an independent correlation between hypovitaminosis D and NAFLD has been reported. In particular, low vitamin D levels have been associated with the histological severity of NAFLD/NASH [18] and with the prevalence of NAFLD among individuals with normal liver enzymes [19]. Overall, a 26 % additional risk for vitamin D deficiency has been reported in NAFLD subjects compared to controls in the only meta-analysis available [20]. A strong epidemiological overlap also exists between NAFLD and hypovitaminosis D prevalence, as both conditions are widely spread among obese dysmetabolic individuals [17, 21]. Vitamin D exerts a direct action on the liver through its specific receptor, VDR, expressed in all hepatic cell populations; notably, its expression negatively correlates with the inflammatory damage in chronic hepatic diseases [22]. Experimental data showed an overall insulin-sensitizing effect of vitamin D via free fatty acids (FFAs) flux modulation and GLUT-4 muscular expression [23], along with its anti-inflammatory, anti-proliferative and anti-fibrotic activities in the liver [24, 25]. Moreover, vitamin D supplementation has been recently demonstrated to reduce the hepatic levels of cytokeratin 18 apoptotic fragment M30 (CK18-M30), a marker of hepatic damage hugely validated in NAFLD/NASH [26, 27] and in rats affected by NASH [28].

Very recently, a prospective small pilot study evaluated the impact of 24-week high-dose oral vitamin D supplementation on liver histology of 12 non-cirrhotic NASH patients, finding no beneficial effects of this treatment on hepatic damage or insulin sensitivity [29]; however, vitamin D effects on hepatic fat content in NAFLD have never been tested. Therefore, the aim of the present study was to assess the efficacy and safety of 24-week oral high-dose vitamin D supplementation in T2D patients affected by NAFLD, specifically assessing hepatic fat.

## Methods

### Role of the funding source

Authors had full access to the trial data. Funders had no role in study design, data collection, analysis, interpretation and decision to publish study findings.

### Study design and participants

This is a monocentric, randomized, double-blind, placebo-controlled trial. Study participants were recruited among patients referring to the Diabetes outpatients’ clinic of Sapienza University of Rome, Italy, for diabetes care. Between March 2012 and September 2014, 65 patients were randomized. To be eligible for the study, patients had to satisfy the following criteria: male or female subjects between 25 and 70 years of age; diagnosis of T2D according to ADA 2009 criteria [30]; presence of fatty liver detected by upper abdominal ultrasound echography (US) and confirmed by magnetic resonance (MRI) in subjects with a clinical suspect of NAFLD (increased serum transaminase levels in absence of known hepatic chronic disease, alanine aminotransferase (ALT) > aspartate aminotransferase (AST), presence of multiple components of metabolic syndrome); negative tests for the presence of hepatitis B surface antigen and antibody to hepatitis C virus. The main exclusion criteria from the study were as follows: history of alcohol abuse (as defined by an average daily consumption of alcohol > 30 g/day in men and > 20 g/day in women), cirrhosis, autoimmune hepatitis and other causes of liver disease (hemochromatosis, Wilson’s disease), chronic enteropathies, advanced renal failure, cancer, hyper/hypoparathyroidism, known hypersensitivity to cholecalciferol or any other excipients, hypercalcemia, hypercalciuria, nephrolithiasis, nephrocalcinosis, ongoing/recent (previous 6 months) supplementation with vitamin D, calcium, multivitamin products, treatment with agents affecting bone and calcium/vitamin D metabolism (anticonvulsants, glucocorticoids, antacids containing aluminum, cholestyramine), UV radiation exposure, pregnancy and lactation, or severe psychiatric illnesses.

### Ethics, consent and permissions

This clinical trial was conducted in accordance with Good Clinical Practice guidelines, and was registered at www.clinicaltrialsregister.eu (number 2011-003010-17). The study protocol was reviewed and approved by the Ethics Committee of Policlinico Umberto I, Sapienza University of Rome and the study was conducted in conformance with the Helsinki Declaration. Written consent was obtained from all patients before the study. The original protocol for the clinical trial (Additional file 1) and the supporting CONSORT checklist (Additional file 2) are provided as supporting information.

### Randomization and masking

Randomization was performed by the statistician following acquisition of participants’ informed consent, through a computer-generated and centrally administered procedure. Patients were randomized 1:1 according to the method of block randomization with a block size of 5. Treatment (cholecalciferol, 25.000 IU/2.5 mL) and placebo were provided in identical vials by an experienced independent pharmacist (Dr. Baiocco E, Rome, Italy); the recommended intake was eight drops a day, equivalent to cholecalciferol 2000 IU/day in the active-treated group, for duration of the study (24 weeks). Patients, investigators, clinical site staff, laboratory staff and radiologists were all masked to treatment assignment throughout the study. Participants were asked to return the drug vials when attending the follow-up visits in order to assess their compliance to study treatment.

### Procedures

After randomization, patients underwent the baseline visit and received the first supply of study medication, as required for 12 weeks. The first follow-up visit took place after 12 weeks and treatment continued for a further 12 weeks; returned vials were checked and collected and then new supplies were provided as required for the last 12 weeks of treatment.

At the baseline, 12- and 24-week visits, study participants underwent a complete work-up including clinical examination, anthropometric measurements and laboratory tests. All medications were carefully recorded at baseline visits and drug alterations regarding antidiabetic agents, anti-hypertensive treatments and statins were not allowed throughout the study.

Weight and height were measured with patients wearing light clothing and no shoes. The body mass index (BMI) was calculated as weight in kilograms divided by the square of the height in meters. Waist circumference was measured midway between the 12th rib and the iliac crest. Blood pressure [systolic (SBP) and diastolic (DBP)] was measured after 5 minutes of rest using an electronic auscultatory blood pressure recorder with an appropriately sized cuff based on the measurement of arm circumference with the patient sitting in the upright position. Three measurements were taken and the average of the second and third measurements was recorded and used in the analyses.

Fasting glycaemia (FBG), glycosylated hemoglobin (HbA1c), total cholesterol, high-density lipoprotein cholesterol (HDL), triglycerides, AST, ALT, gamma-glutamyl transpeptidase (γ-GT) and C-reactive protein (CRP) were measured by standard laboratory methods. Fasting blood insulin (FBI) was assessed by radio-immuno-assay (PANTEC s.r.l., Italy; intra- and inter-assay coefficients of variation < 5 %). Serum FFAs were measured by standard colorimetric methods and circulating adiponectin levels by enzyme-linked immunosorbent assay (Tema Ricerca s.r.l., Italy; intra- and inter-assay coefficients of variation = 5 %). As non-invasive biomarkers of hepatic damage and fibrosis, we measured serum CK18-M30 concentrations by Human Cytokeratin 18-M30 ELISA kit (Cusabio^®^, intra- and inter-assay coefficients of variation < 8 %) and circulating N-terminal Procollagen III Propeptide (P3NP) levels [31] by Human PIIINP ELISA kit, Elabscience™ (intra- and inter-assay coefficients of variation < 10 %). Serum 25(OH) vitamin D concentration (25(OH)D) was measured as an indicator of vitamin D status [32] by a validated colorimetric method (LAISON, DiaSorin) and then adjusted on the basis of the sampling period, as described elsewhere [33]. Low-density lipoprotein cholesterol (LDL) values were calculated using the Friedewald formula. The homeostasis model assessment of insulin resistance (HOMA-IR) and insulin secretion (HOMA-β%) and the quantitative insulin sensitivity check index (QUICKI) were calculated as previously described [34]; the adipose tissue (AT) insulin-resistance was quantified by the ADIPO-IR index [35]. Fatty Liver Index (FLI) was used as a clinical correlate of NAFLD [36]. Liver US scanning was performed to assess the presence of hepatic steatosis by an Esaote Medica apparatus equipped with a convex 3.5 MHz probe.

All MRIs were performed by the same operator, unaware of treatment group and blinded to laboratory values, at the screening visit and within two weeks from the 24-week visit, using a 1.5-T magnet (Magnetom Avanto, Siemens Medical Systems, Erlangen, Germany) equipped with a phased-array surface coil and a spine array coil. Image acquisition was performed in the axial plane during an end-expiratory breath-hold using a sensitivity encoding (SENSE) technique in order to reduce the overall acquisition time to approximately 15 sec. The hepatic fat fraction (HFF) was obtained by using a two-dimensional spoiled GRE acquired on the axial plane. To minimize T1 effects, a low flip angle (10°) was used at a repetition time of 150 msec. To estimate fat-water signal interference and T2* effects, three echoes were obtained at serial opposed-phase (OP) and in-phase (IP) echo times (2.3, 4.7, 6.9 msec). Other parameters applied were: section thickness (5 mm), matrix size (256 × 182) and field of view (35 × 40 cm) [37]. HFF was calculated from the mean of the two in-phase sequences (IP correct) subtracted to the out-of-phase sequence and then divided to the “2* IP correct” sequence. Eight different ROIs measuring 2 cm^2^ were drawn, one for each hepatic segment within the liver, avoiding areas with vessels, motion artifacts and partial volume effects; ROIs were placed at anatomically matched locations on paired images by using a co-registration tool available on the picture archiving and communication system workstation. Finally, mean ± SD HFF was calculated for each patient. For visceral and subcutaneous adipose tissue area quantification (VAT, SAT; cm^2^) a 3D GRE T1-weighted VIBE sequence on axial plane modified by DIXON was acquired (TR, 4.7 msec; TE, 2.3 msec; flip-angle, 10 °C; matrix, 256 × 192 mm; section thickness, 5 mm, reconstructed 2.5 mm; intersection gap, 0). The fat-only datasets were transferred to personal computers for the analysis using a commercially available software (Slice-O-Matic; Tomovision Inc., Montreal, Canada) and data were calculated from AT area at L1-L2, L2-L3, L3-L4 and L4-L5 levels; a free-form ROI and manual threshold were used to select fat tissue within VAT and SAT slides. Means ± SD basal and 24-week VAT and SAT areas were then calculated in each patient for statistical purposes.

The cardiovascular evaluation was carried out at the baseline and 24-week visit. Carotid longitudinal ultrasound was performed to measure the intima-media thickness (IMT) of both sides, 1 cm proximally to the carotid bulb. Three IMT measurements were obtained and then the mean was calculated; the average value between right and left IMT was used for the statistical analyses. US assessment of endothelial dependent and independent flow-mediated dilatation (FMD) of brachial artery was investigated by a 7.5-MHz linear array transducer ultrasound system (Siemens) equipped with electronic calipers, vascular software for two-dimensional imaging, color and spectral Doppler, and internal electrocardiogram; FMD was expressed as a change in post-stimulus diameter (percentage of the baseline diameter). The ankle-brachial index (ABI) was calculated as the ratio of ankle and brachial SBP measured separately for the right and left sides, then an average value was calculated for statistical purposes.

### Primary and secondary outcomes

The primary outcome was the reduction of MRI-measured HFF from baseline to 24 weeks. As additional indicators of hepatic injury in NAFLD we considered the changes in transaminases, CK18-M30, P3NP levels and FLI from baseline to 24 weeks. Secondary outcomes were the metabolic profile’s improvement, as assessed by changes in FBG, FBI, HbA1C, total, HDL and LDL cholesterol, triglycerides, FFAs, HOMA-IR, QUICKI, ADIPO-IR, HOMA-β and body fat distribution (VAT and SAT areas), and the modification of endothelial-cardiovascular parameters SBP, DBP, ABI, IMT, FMD and circulating CRP levels from baseline to the end of the study. Adverse events were recorded at each visit.

### Statistical analysis

This is the first randomized controlled trial aiming to investigate the effect of oral vitamin D supplementation on NAFLD. Considering a treated/controls ratio of 1:1, an estimated HFF reduction of 50 % in the active treated group and of 10 % in the placebo group, together with a drop-out rate of 10 %, we needed to enroll 24 + 10 % = 27 patients in each group, with a power of 80 % and a sensitivity of 95 %. Differences between the treated and control groups were evaluated by non-parametric tests. Linear regression analyses, adjusted for baseline values, were performed to assess the mean difference between the intervention and the placebo groups after 24 weeks (mean difference is reported as β, along with the 95 % confidence interval); SPSS version 23 was used to perform all the analyses.

## Results

**Fig. 1:**
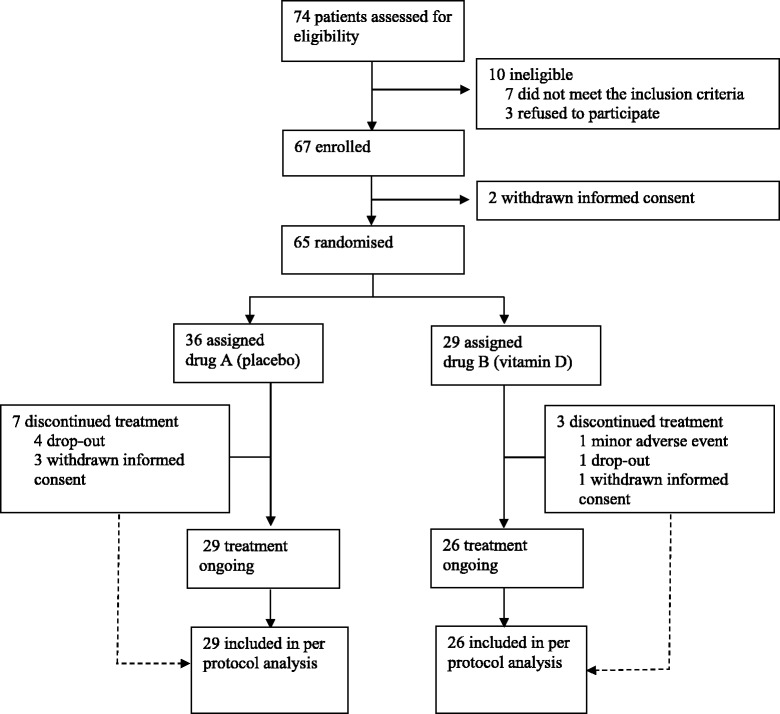
Trial profile

**Fig. 2:**
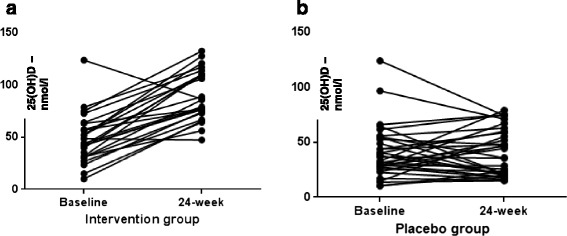
Comparison between serum 25(OH) D levels in intervention (**a**) and placebo (**b**) group. Wilcoxon’s test for paired samples applied

**Fig. 3:**
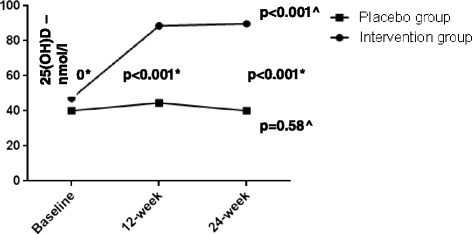
Serum 25(OH) D levels in intervention versus placebo group. * Independent samples U Mann–Whitney’s test; ^ Multiple dependent comparisons Friedman’s test

Of the 74 patients screened for eligibility, 65 met the inclusion criteria and were recruited in our study. Of these, 36 were allocated to group A (placebo) and 29 to group B (cholecalciferol 2000 IU/day); in total, 84.6 % of study population (*n* = 29 in placebo and *n* = 26 in treatment group) completed the study (Fig. 1). Four patients withdrew their informed consent before study conclusion and four patients were lost at follow-up. As per the safety profile, no major adverse events occurred during the study, one patient in the treatment group referred new-onset mild glossitis after 3 weeks from randomization and discontinued the study treatment. The mean age of all patients was 58.7 ± 9.9 years, with mean diabetes’ duration of 6.5 ± 5.5 years; males represented 70 % of the study population. Subgroups did not differ for any parameters, nor for ongoing medications (Tables 1 and 2). At baseline, 92 % of the study population had sub-optimal serum 25(OH)D levels (<75 nmol/L) [38] and 67 % had hypovitaminosis D (<50 nmol/L). Circulating 25(OH)D concentration significantly increased after oral cholecalciferol supplementation (Fig. 2), and this goal was achieved already after 12 weeks of treatment (Fig. 3); at 24 weeks, 96 % of active treatment patients reached sufficient vitamin D balance (≥50 nmol/L) and 71 % showed optimal 25(OH)D levels (≥75 nmol/L). As expected, no significant changes in serum 25(OH)D levels occurred in the placebo group throughout the study (Fig. 3).

**Table 1:** Clinical and biochemical characteristics of study population according to group of treatment

	25(OH)D group baseline (*n* = 26)	Placebo group baseline (*n* = 29)
Age (years)	57.4 ± 10.7	59.8 ± 9.1
Gender (%M)	70 %	60 %
T2D duration (years)	5.9 ± 5.8	6.3 ± 5.4
25(OH)D (nmol/L)	48.15 ± 23.7	40.14 ± 23.9
HFF (%)	7.6 ± 5.7	6.8 ± 5.5
BMI (kg/m^2^)	29.3 ± 4.4	30.8 ± 4.5
Waist circumference (cm)	100.6 ± 15.2	105.2 ± 12.1
SBP (mmHg)	129.7 ± 16.8	132.2 ± 17.4
DBP (mmHg)	79.2 ± 8.9	82.7 ± 10.7
Total cholesterol (mg/dL)	167.7 ± 37.5	181.6 ± 39.5
HDL-C (mg/dL)	50.3 ± 16.4	49.3 ± 13.2
LDL-C (mg/dL)	93 ± 33.6	105.2 ± 34.5
Triglycerides (mg/dL)	131.5 ± 72.7	133.5 ± 43.8
FBG (mg/dL)	125.3 ± 36.8	135.1 ± 39.9
HbA1c (%/mmol/mol)	6.36 ± 0.9/46 ± 8	6.6 ± 1/48 ± 8
AST (IU/L)	24.12 ± 11.8	23.8 ± 14.6
ALT (IU/L)	31.7 ± 17.1	32.4 ± 26.2
γ-GT (IU/L)	45.3 ± 56.4	35.8 ± 33.3
AST/ALT	0.84 ± 0.3	0.83 ± 0.26
FFAs (μmol/L)	464.6 ± 224.5	519.9 ± 218.4
CK18-M30 (mIU/mL)	212.2 ± 128.2	212.1 ± 155.2
P3NP (pg/mL)	1210.5 ± 1028.6	833.9 ± 955.4
FBI (μU/L)	12 ± 5.1	12.7 ± 5.8
FLI	56.82 ± 26.4	67.7 ± 23.7
HOMA-IR	3.57 ± 1.9	3.87 ± 1.6
HOMA-β%	89.6 ± 63	83.8 ± 63.7
QUICKI	0.33 ± 0.03	0.32 ± 0.02
ADIPO-IR	5.1 ± 3.5	6.3 ± 4.7
CRP (mg/dL)	3.1 ± 3.1	3.6 ± 4.8
Adiponectin (ng/mL)	6.37 ± 3.7	6.4 ± 3.2
VAT area (cm^2^)	195.9 ± 78.2	191.4 ± 65.9
SAT area (cm^2^)	229 ± 28.4	258.01 ± 123.9
VAT/SAT ratio	1.07 ± 0.6	1.07 ± 0.62
FMD (%)	5.04 ± 4.5	4.5 ± 3.6
ABI	1.14 ± 0.19	1.1 ± 0.13
IMT (mm)	0.91 ± 0.25	0.87 ± 0.19

Data are presented as mean ± SD, unless indicated otherwise

*T2D* type 2 diabetes, *HFF* hepatic fat fraction, *BMI* body mass index, *SBP* systolic blood pressure, *DBP* diastolic blood pressure, *FBG* fasting blood glucose, *FBI* fasting blood insulin, *FLI* fatty liver index, *CRP* C reactive protein, *VAT* visceral adipose tissue, *SAT* subcutaneous adipose tissue, *FMD* flow-mediated dilatation, *ABI* ankle-brachial index, *IMT* intima-media thickness

**Table 2:** Ongoing therapies in study population according to group of treatment

	25(OH)D group baseline (*n* = 26)	Placebo group baseline (*n* = 29)
Insulin treatment (% patients)	16 %	18 %
Number of oral antidiabetic agents (% patients)		
0	11 %	16 %
1	50 %	43 %
2	29 %	30 %
3	10 %	11 %
Statins treatment (% patients)	68 %	57 %
Anti-hypertensive treatment (% patients)	76 %	75 %

Data are presented as mean ± SD, unless indicated otherwise

Regarding the primary endpoint, changes in HFF from baseline to 24 weeks did not differ significantly between the two study groups (β, 0.63; 95 % CI, –1.6 to 2.8; *P* = 0.57). Similarly, none of the other hepatic indicators, such as AST, ALT, γ-GT, AST/ALT, CK18-M30, P3NP and FLI, showed significant changes between the active treated group and placebo after 24 weeks (Table 3). Vitamin D supplementation was demonstrated to have a neutral effect on both metabolic profile and cardiovascular parameters, as no significant differences between the groups were found in the indicators of metabolic control (FBG, HbA1C, lipid profile), insulin resistance (HOMA-IR, HOMA-β%, QUICKI, FBI, FFAs), body fat distribution (SAT, VAT, VAT/SAT area), CRP, IMT and FMD. Changes in ABI at the end of the study differed between treated and placebo group (β, –0.10; 95 % CI, –0.18 to –0.01; *P* = 0.03) but this difference was not significant after correcting for multiple comparisons (data not shown).

**Table 3:** Comparison of characteristics before and after study treatment in the vitamin D supplementation and placebo groups

	25(OH)D group baseline	25(OH)D group 24-week	Placebo group baseline	Placebo group 24-week	Adjusted β (95 % CI)*	*P* value
25(OH)D (nmol/L)	43.1 (31.1–58.5)	85.8 (73–110)	37.1 (27.3–51.6)	40 (20.8–60.5)	48.6 (35.8 to 61.3)	<0.001*
HFF (%)	6.8 (3.9–14.8)	7 (4.4–12.9)	5.9 (3.2–11.2)	5.3 (3.2–9.7)	0.63 (–1.6 to 2.8)	0.57
BMI (kg/m^2^)	27.9 (27.2–31.8)	27.9 (26.8–31.4)	30.2 (27–33.5)	30.8 (27.6–32.6)	0.05 (–1.3 to 1.4)	0.94
Waist circumference (cm)	99 (92–106)	94.2 (99.5–107.6)	105 (95–110)	105 (96.5–109)	0.86 (–2.8 to 4.5)	0.64
SBP (mmHg)	130 (120–140)	130 (120–140)	130 (120–140)	130 (120–150)	–2.6 (–12.3 to 6.9)	0.58
DBP (mmHg)	80 (70–90)	80 (75–90)	80 (75–90)	80 (80–90)	–1.38 (–6.8 to 4)	0.61
Total cholesterol (mg/dL)	176 (147–195)	168.5 (144–194)	179 (150–209)	162 (146–194)	–5.7 (–22.3 to 10.8)	0.49
HDL-C (mg/dL)	50 (34–59)	48 (42–54)	47.5 (40.2–54)	49 (41–61)	–0.46 (–5.1 to 4.2)	0.84
LDL-C (mg/dL)	92 (63–116)	88 (65.5–103.7)	94.3 (78–129)	85 (73–105)	–4.6 (–19.5 to 10.3)	0.53
Triglycerides (mg/dL)	100 (65–181)	116.5 (87–176)	128.5 (104.5–164)	116 (91.5–154.2)	–0.24 (–42.5 to 42)	0.99
FBG (mg/dL)	126 (103–148)	115 (102.114)	124 (107.5–154)	127 (105–160)	2.5 (–18.9 to 23.9)	0.48
HbA1c (%/mmol/mol)	6.3 (5.9–6.7)/45(41–50)	6 (5.6–6.9)/42 (38–52)	6.5 (6–7.2)/48(38–55)	6.4 (6–7.3)/46 (38–56)	–0.05 (–0.42 to 0.33)	0.80
AST (IU/L)	20 (17–30)	20.5 (16–25.7)	20.5 (16–27)	22 (17–30)	–2.4 (–5.7 to 0.79)	0.13
ALT (IU/L)	26 (18–48)	29 (20–37.5)	27 (19.5–40)	28.5 (21–40)	–4.6 (–11.9 to 2.5)	0.20
γ-GT (IU/L)	29 (19–53)	28 (19.5–43)	22.5 (16–39)	23 (17–40.5)	–0.95 (–6.1 to 4.2)	0.71
AST/ALT	0.83 (0.62–1)	0.8 (0.6–1)	0.77 (0.67–0.92)	0.72 (0.61–0.92)	0.02 (–0.11 to 0.06)	0.60
CK18-M30 (mIU/mL)	186.7 (139–240.4)	170.3 (102.1–271.9)	212.6 (66–315.9)	130.3 (85–234.6)	0.08 (–63 to 114.7)	0.56
P3NP (pg/mL)	1334.9 (166.8–1981)	1229.5 (327.6–1924)	464.6 (66.4–1270.9)	390.1 (116.2–1290)	0.19 (–23.7 to 738.4)	0.07
FFAs (μmol/L)	13.6 (8.8–19.7)	6.5 (4.2–8)	14.1 (8.9–19.7)	4.9 (3.2–8.1)	21 (–10.8 to 15)	0.75
FBI (μU/L)	11.7 (7.4–15.8)	11.5 (8.8–17.2)	13 (8.3–16)	13.1 (9.5–19)	–1.2 (–3.7 to 1.3)	0.35
FLI	68.6 (33–83.2)	54.5 (35.5–79.8)	79.8 (50.2–89)	70.5 (57–84.3)	–5.3 (–15.9 to 5.3)	0.32
HOMA-IR	3.9 (1.9–5.9)	3.4 (1.9–5.6)	4 (2.5–5.5)	4 (2.8–6.1)	0.01 (–1.3 to 1.3)	0.98
HOMA-β%	71.3 (50.3–114.8)	68 (51.8–97.5)	67.3 (42–126.3)	71 (50–116)	–18.7 (–49.1 to 11.7)	0.22
QUICKI	0.31 (0.3–0.34)	0.32 (0.3–0-34)	0.31 (0.3–0.33)	0.31 (0.29–0.33)	0.005 (–0.008 to 0.018)	0.40
ADIPO-IR	5.4 (2.5–9.5)	2.4 (1.3–3.7)	5.7 (2.8–7.9)	2.8 (1.5–4.3)	0.013 (–1.66 to 1.68)	0.98
CRP (mg/dL)	3 (0.25–6.6)	0.8 (0.15–1.6)	1.3 (0.6–4)	0.5 (0.1–3.5)	–0.04 (–1.6 to 1.5)	0.96
Adiponectin (ng/mL)	5.3 (3.3–9.6)	12.7 (9.9–22.8)	5.7 (3.9–8.9)	12.2 (9.5–16.4)	5.2 (–3.57 to 14)	0.24
VAT area (cm^2^)	173.4 (138.5–251)	183 (130–322)	190 (127.3–238)	188.7 (125–265.4)	6.1 (–45.9 to 58.1)	0.81
SAT area (cm^2^)	195.7 (132.5–340)	210.7 (115–366)	222.6 (169–371)	249.2 (188–392)	–21.9 (–74.07 to 30.08)	0.4
VAT/SAT ratio	0.93 (0.48–1.6)	0.93 (0.59–1.5)	0.81 (0.52–1.2)	0.89 (0.39–1.2)	0.08 (–0.18 to 0.28)	0.54
FMD (%)	2.9 (0.7–8.1)	2.5 (0.9–4.7)	4.4 (2.1–6.4)	4.3 (1.7–6.2)	–1.8 (–4.6 to 1.02)	0.20
ABI	1.1 (1–1.2)	1.04 (0.9–1.1)	1.13 (1–1.2)	1.1 (1–1.1)	–0.10 (–0.18 to –0.01)	0.03*
IMT (mm)	0.8 (0.72–0.95)	0.81 (0.7–1)	0.8 (0.74–1)	0.84 (0.7–1)	–0.04 (–0.14 to 0.04)	0.33

Data are presented as median (25°–75° percentile), unless indicated otherwise, significance between groups was assessed by linear regression analysis adjusted for the baseline value

*Statistically significant

*T2D* type 2 diabetes, *HFF* hepatic fat fraction, *BMI* body mass index, *SBP* systolic blood pressure, *DBP* diastolic blood pressure, *FBG* fasting blood glucose, *FBI* fasting blood insulin, *FLI* fatty liver index, *CRP* C reactive protein, *VAT* visceral adipose tissue, *SAT* subcutaneous adipose tissue, *FMD* flow-mediated dilatation, *ABI* ankle-brachial index, *IMT* intima-media thickness

In order to investigate whether vitamin D supplementation may improve NAFLD exclusively in patients with hypovitaminosis D at the baseline, we performed an ancillary analysis to compare changes in the primary outcome from the baseline to 24-week including only patients with basal 25(OH)D <50 nmol/L, but again changes in HFF were not significantly different between treatment and placebo group in this subpopulation (β, 2.1; 95 % CI, −0.66 to 4.78, *P* = 0.13); similar results were found with all other markers of liver involvement [AST: β, –0.4; 95 % CI, –4.57 to 3.78; *P* = 0.85; ALT: β, 0.5; 95 % CI, –0.9 to 8.2; *P* = 0.89; γ-GT: β, 0.92; 95 % CI, –6 to 7.8, *P* = 0.79; AST/ALT: β, 0.02; 95 % CI, –0.08 to 0.11; CK-M30: β, 0.08; 95 % CI, –87.6 to 150.7, *P* = 0.59; P3NP: β, 0.25; 95 % CI, –17.2 to 71,081; *P* = 0.06; FLI: β, –5.7; 95 % CI, –20.3 to 8.8, *P* = 0.42].

## Discussion

This is the first randomized, double-blind, placebo-controlled trial of high-dose vitamin D oral supplementation performed in T2D patients with NAFLD. This trial showed that a nutraceutical intervention based on high-dose vitamin D oral supplementation has no effect on hepatic fat content in T2D patients affected by NAFLD – 24-week cholecalciferol supplementation did not improve either transaminases levels or the serum levels of biomarkers specific for hepatic injury and fibrogenesis such as CK18-M30 and P3NP. Similarly, clinical surrogates of liver impairment in the course of NAFLD, such as AST/ALT ratio and FLI, did not show significant changes after vitamin D supplementation. As we specifically aimed to study the effect of vitamin D on fatty liver in diabetic patients, we also tested the hypothesis of an involvement of vitamin D in modulating insulin-resistance, metabolic profile and glycemic control in these subjects, but none of these parameters significantly changed after 24-week vitamin D treatment in comparison with the placebo group. Likewise, vitamin D supplementation did not induce any specific effect on endothelial function and subclinical atherosclerosis. Furthermore, as not all the study participants displayed low circulating 25(OH)D levels at the baseline, we postulated that vitamin D supplementation could exert favorable effects on NAFLD only in patients affected by hypovitaminosis D, but the ancillary analyses did not confirm this hypothesis, as we observed a similar response in patients with normal or reduced 25(OH)D at the baseline. Previously, Sharifi et al. [39] investigated the effect of twice a month 16-week cholecalciferol supplementation on aminotransferases, insulin resistance and inflammatory profile in non-diabetic subjects selected on the basis of US-detected fatty liver and upper-than-normal ALT levels, but no effects were shown compared to placebo. Notably, a significant decrease in the levels of hsCRP and malondialdehyde (a marker of lipid peroxidation) was found in the subjects treated with vitamin D. In this study, the intervention was limited to 4 months and, although the authors aimed to test a possible effect on insulin resistance, the study was performed just in non-diabetic patients. Furthermore, US did not allow performing of a reliable and validated quantification of hepatic fat content changes before and after study treatment. Moreover, in a recent study, high-dose oral vitamin D3 supplementation (25,000 IU/week) over 24 weeks had no impact on liver histology, liver biochemistry, insulin resistance or adipocytokine profile in 12 non-diabetic patients with biopsy-proven NASH [29].

Our trial has a number of strengths. It is the first study investigating vitamin D effects on hepatic fat content in T2D subjects as measured by MRI. In addition, we evaluated serum transaminases, CK18-M30, P3NP, AST/AST ratio and FLI as possible indicators of hepatic damage in presence of NAFLD and their changes before and after 24-week cholecalciferol supplementation. The study population was recruited in the same diabetes center and, therefore, all the outcome measurements have been centralized by definition. Since vitamin D effects on NAFLD were tested in T2D patients, we evaluated, as secondary endpoints, the influence of vitamin D treatment on systemic and AT insulin resistance, insulin secretion and glycemic control. Along with the metabolic profile, we were able to provide a description of the effect of 24-week cholecalciferol treatment on surrogate markers of subclinical atherosclerosis and endothelial dysfunction, allowing to extensively investigate the impact of high-dose oral cholecalciferol supplementation on cardiovascular risk in patients with both NAFLD and T2D. Furthermore, 96 % of the active-treated group reached vitamin D sufficiency at the end of the study, showing both the optimal compliance to study treatment and the adequacy of the cholecalciferol dosage provided.

We are aware that the dimension of this study population is not particularly large. However, the final sample size was greater than the one in the only trial published so far on non-diabetics with NAFLD [39] and, despite the relatively high rate of dropouts, was properly powered. Indeed, at the light of the average HFF < 10 % observed in patients referring to our Center for clinical evaluations, an HFF reduction of 50 % was considered as a clinically relevant goal, allowing to reach a steatosis-free status in the majority of study participants. In addition, the study population has been extensively phenotyped and well matched for all the features of NAFLD, diabetes and metabolic syndrome.

One limitation of our study is the lack of liver biopsies, which did not allow us to evaluate histological changes in our cohort. However, as our population exhibited relatively mild hepatic disease, the surrogate endpoints assessed by MRI, along with the measurement of serum CK-18 and P3NP levels as validated biomarkers of hepatic damage and fibrogenesis in the course of NAFLD [26, 27, 31], can be considered suitable for this phase of the study.

Despite some studies demonstrating the association between hypovitaminosis D and NAFLD/NASH [18–20] and the direct effect of vitamin D in modulating hepatic inflammation, fibrosis and insulin-resistance both in vivo and in vitro [22–25, 28], very recently, the existence of an independent relationship between biopsy-proven NASH and circulating low 25(OH)D levels has been confuted by two independent studies performed in subjects with different metabolic phenotypes [40, 41].

Although the active treated group almost doubled the mean 25(OH)D levels after just 12-week supplementation, our study did not meet the primary endpoint of showing a reduction of hepatic fat content in NAFLD patients undergoing 24-week high-dose oral cholecalciferol supplementation. Indeed, it is possible to speculate that either the period of exposure to normal-optimal 25(OH)D concentration was not enough for modifying the hepatic fat content and the clinical/biochemical indicators of hepatic involvement in NAFLD, or the link between vitamin D and NAFLD could be appreciated only in specific sub-populations of patients with fatty liver. As we aimed specifically to assess the efficacy of vitamin D supplementation on liver steatosis in T2D patients, the study population was selected among T2D patients referring to our Diabetes outpatients’ clinic for routine diabetes care. Likewise, this setting led to recruitment of subjects with milder NAFLD levels than those detectable in patients purposely referring to Hepatology clinics for the treatment of liver diseases. Indeed, we cannot rule out that a certain effect of oral vitamin D may be appreciated in patients with more severe NAFLD and NASH. Considering the overall strong rationale behind the favorable cost/benefit ratio and the safety of oral vitamin D supplementation, further randomized controlled studies, with a longer period of intervention and performed in different populations, are warranted before definitively excluding a role of vitamin D in NAFLD treatment.

On the other hand, it is also plausible to hypothesize that, despite hypovitaminosis D representing an independent risk factor for NAFLD, once this condition is established, late vitamin D supplementation may not be capable of reverting the negative effects of its prolonged deficiency on liver parenchyma. Therefore, strategies preventing hypovitaminosis D in the general population and, in particular, in dysmetabolic patients at increased risk of NAFLD could result in better outcomes than intervention studies performed after NAFLD diagnosis.

## Conclusions

This study demonstrated for the first time that a nutraceutical intervention based on 24-week oral vitamin D supplementation did not improve hepatic steatosis or metabolic/cardiovascular parameters in T2D patients with NAFLD. Hepatic steatosis still remains a widespread condition without an appropriate therapy. Studies with longer intervention periods, even in subjects at high risk of NAFLD, are warranted for exploring the effect of long time exposure to vitamin D.

## Abbreviations

25(OH)D, 25(OH) vitamin D; ABI, ankle-brachial index; ALT, alanine aminotransferase; AST, aspartate aminotransferase; AT, adipose tissue; BMI, body mass index; CK18-M30, Human Cytokeratin 18-M30; CRP, C-reactive protein; DBP, diastolic blood pressure; FBG, fasting glycaemia; FBI, fasting blood insulin; FFAs, free fatty acids; FLI, fatty liver index; FMD, flow-mediated dilatation; HbA1c, glycosylated hemoglobin; HDL, high-density lipoprotein cholesterol; HFF, hepatic fat fraction; HOMA-IR, homeostasis model assessment of insulin resistance; HOMA-β%, homeostasis model assessment of insulin secretion; IMT, intima-media thickness; LDL, low-density lipoprotein cholesterol; MRI, magnetic resonance; NAFLD, nonalcoholic fatty liver disease; NASH, nonalcoholic steatohepatitis; NASH, non-alcoholic steatohepatitis; P3NP, N-terminal Procollagen III Propeptide; QUICKI, quantitative insulin sensitivity check index; SAT, subcutaneous adipose tissue; SBP, systolic blood pressure; T2D, type 2 diabetes; US, ultrasound echography; VAT, visceral adipose tissue; VDR, vitamin D receptor; γ-GT, gamma-glutamyl transpeptidase.

## Additional files

Additional file 1: Study protocol. (PDF 134 kb) Additional file 2: CONSORT 2010 checklist. (DOC 216 kb)
