# Supplementary material for: No effects of oral vitamin D supplementation on non-alcoholic fatty liver disease in patients with type 2 diabetes: a randomized, double-blind, placebo-controlled trial
Source: BMC Med. 2016 Jun 29;14:92. doi: 10.1186/s12916-016-0638-y (PMC4926287; doi:10.1186/s12916-016-0638-y)
Supplement: Additional file 1: — Study protocol. (PDF 134 kb) [file 12916_2016_638_MOESM1_ESM.pdf]

www.clinicaltrialsregister.eu

Clinical trials for 2011-003010-17

Summary

EudraCT Number: 2011-003010-17

Sponsor's Protocol Code Number: -

National Competent Authority: Italy - Italian Medicines Agency

Clinical Trial Type: EEA CTA

Trial Status: Ongoing

Date on which this record was first entered in the EudraCT database: 2012-03-07

Link: https://www.clinicaltrialsregister.eu/ctr-search/trial/2011-003010-17/IT/

A. Protocol Information

A.1 Member State Concerned: Italy - Italian Medicines Agency

A.2 EudraCT number: 2011-003010-17

A.3 Full title of the trial: Randomized double-blind intervention trial to assess the efficacy of vitamin D3 supplementation vs placebo in reducing hepatic steato-inflammation and cardio-metabolic risk profile in patients affected by type 2 diabetes and non-alcoholic fatty liver disease (NAFLD/NASH)

A.3 Full title of the trial (it): STUDIO DI INTERVENTO RANDOMIZZATO CONTRO PLACEBO PER VALUTARE LA EFFICACIA DELLA SUPPLEMENTAZIONE ORALE CON VITAMINA D3 NEL RIDURRE LA STEATO-INFIAMMAZIONE EPATICA E NEL MODIFICARE IL PROFILO DI RISCHIO CARDIO-METABOLICO IN ADULTI DIABETICI AFFETTI DA STEATOSI/STEATOEPATITE NON ALCOOL-CORRELATA (NAFLD/NASH)

A.3.1 Title of the trial for lay people, in easily understood, i.e. non-technical, language: Randomized double-blind intervention trial to assess the efficacy of vitamin D3 supplementation vs placebo in reducing hepatic steato-inflammation and cardio-metabolic risk profile in patients affected by type 2 diabetes and non-alcoholic fatty liver disease (NAFLD/NASH)

A.3.1 Title of the trial for lay people, in easily understood, i.e. non-technical, language (it): STUDIO D’INTERVENTO RANDOMIZZATO CONTRO PLACEBO PER VALUTARE L’EFFICACIA DELLA SUPPLEMENTAZIONE ORALE CON VITAMINA D3 NEL RIDURRE LA STEATOSI E L'INFIAMMAZIONE NEL FEGATO E I FATTORI DI RISCHIO CARDIOVASCOLARI IN ADULTI DIABETICI AFFETTI DA STEATOSI/STEATOEPATITE NON ALCOLICA (NAFLD/NASH)

A.4.1 Sponsor's protocol code number: -

A.7 Trial is part of a Paediatric Investigation Plan: No

A.8 EMA Decision number of Paediatric Investigation Plan:

B. Sponsor Information

Sponsor 1

B.1.1 Name of Sponsor: AZIENDA UNIVERSITARIA POLICLINICO UMBERTO I DI ROMA

B.1.3.4 Country: Italy

B.3.1 and B.3.2 Status of the sponsor: Non-Commercial

B.4 Source(s) of Monetary or Material Support for the clinical trial:

B.4.1 Name of organisation providing support: Fondi PRIN 2008

B.4.2 Country: Italy

B.5 Contact point designated by the sponsor for further information on the trial

B.5.1 Name of organisation: Sapienza Universita' di Roma

B.5.2 Functional name of contact point: Dott.ssa Maria Gisella Cavallo

B.5.3 Address

B.5.3.1 Street Address: Viale del Policlinico 155

B.5.3.2 Town/ city: Roma

B.5.3.3 Post code: 00161

B.5.3.4 Country: Italy

B.5.4 Telephone number: 3207172510

B.5.5 Fax number: 0649974698

B.5.6 E-mail: gisella.cavallo@uniroma1.it

D. IMP Identification

D.IMP: 1

D.1.2 and D.1.3 IMP Role: Test

D.2 Status of the IMP to be used in the clinical trial

D.2.1 IMP to be used in the trial has a marketing authorisation: Yes

D.2.1.1.1 Trade name: DIBASE*OS GTT 10ML 10000UI/ML

D.2.1.1.2 Name of the Marketing Authorisation holder: ABIOGEN PHARMA SpA

D.2.1.2 Country which granted the Marketing Authorisation: Italy

D.2.5 The IMP has been designated in this indication as an orphan drug in the Community: No

D.2.5.1 Orphan drug designation number:

D.3 Description of the IMP

D.3.4 Pharmaceutical form: Oral drops, solution

D.3.4.1 Specific paediatric formulation: No

D.3.7 Routes of administration for this IMP:

Oral use

D.3.8 to D.3.10 IMP Identification Details (Active Substances)

D.3.8 INN - Proposed INN: NA

D.3.9.1 CAS number: NA

D.3.9.2 Current sponsor code: NA

D.3.9.3 Other descriptive name: NA

D.3.9.4 EV Substance Code: NA

D.3.10 Strength

D.3.10.1 Concentration unit: U unit(s)

D.3.10.2 Concentration type: up to

D.3.10.3 Concentration number: 2000

D.3.11 The IMP contains an

D.3.11.1 Active substance of chemical origin: Yes

D.3.11.2 Active substance of biological/ biotechnological origin (other than Advanced Therapy IMP (ATIMP): No

D.3.11.3 Advanced Therapy IMP (ATIMP): No

D.3.11.3.1 Somatic cell therapy medicinal product: No

D.3.11.3.2 Gene therapy medical product: No

D.3.11.3.3 Tissue Engineered Product: No

D.3.11.3.4 Combination ATIMP (i.e. one involving a medical device): No

D.3.11.3.5 Committee on Advanced therapies (CAT) has issued a classification for this product: No

D.3.11.4 Combination product that includes a device, but does not involve an Advanced Therapy: No

D.3.11.5 Radiopharmaceutical medicinal product: No

D.3.11.6 Immunological medicinal product (such as vaccine, allergen, immune serum): No

D.3.11.7 Plasma derived medicinal product: No

D.3.11.8 Extractive medicinal product: No

D.3.11.9 Recombinant medicinal product: No

D.3.11.10 Medicinal product containing genetically modified organisms: No

D.3.11.11 Herbal medicinal product: No

D.3.11.12 Homeopathic medicinal product: No

D.3.11.13 Another type of medicinal product: Yes

D.3.11.13.1 Other medicinal product type: SUPPLEMENTAZIONE VITAMINICA

D.8 Information on Placebo

E. General Information on the Trial

E.1 Medical condition or disease under investigation

E.1.1 Medical condition(s) being investigated: Patients affected by type 2 diabetes and non alcoholic fatty liver disease- non alcoholic steato-hepatitis (NAFLD/NASH)

E.1.1 Medical condition(s) being investigated (it): Pazienti affetti da diabete di tipo 2 e steatosi-steatoepatite non alcool-correlata (NAFLD/NASH)

E.1.1.1 Medical condition in easily understood language: Patients affected by type 2 diabetes and non alcoholic fatty liver disease- non alcoholic steato-hepatitis (NAFLD/NASH)

E.1.1.1 Medical condition in easily understood language (it): Pazienti affetti da diabete di tipo 2 e steatosi-steatoepatite non alcool-correlata (NAFLD/NASH)

E.1.1.2 Therapeutic area: Diseases [C] - Nutritional and Metabolic Diseases [C18]

MedDRA Classification

E.1.2 Medical condition or disease under investigation:

E.1.2 Version: 14.1

E.1.2 Level: SOC

E.1.2 Classification code: 10027433

E.1.2 Term: Metabolism and nutrition disorders

E.1.2 System Organ Class: 10027433 - Metabolism and nutrition disorders

E.1.3 Condition being studied is a rare disease: No

E.2 Objective of the trial

E.2.1 Main objective of the trial: To evaluate the efficacy of oral supplementation with cholecalciferol in improving liver steato-inflammation in patients with type 2 diabetes mellitus and diagnosis of NAFLD or NASH.

E.2.1 Main objective of the trial (it): Valutare l'efficacia della supplementazione orale con colecalciferolo nel migliorare la steato-infiammazione epatica in pazienti con diabete mellito di tipo 2 e diagnosi di NAFLD o NASH.

E.2.2 Secondary objectives of the trial: To evaluate the efficacy of oral supplementation with cholecalciferol in improving cardio-metabolic risk profile (insulin-sensitivity, lipids, sistemic inflammation, glucose tolerance, body fat distribution) in patients with type 2 diabetes mellitus and diagnosis of NAFLD or NASH.

E.2.2 Secondary objectives of the trial (it): Valutare l'efficacia della supplementazione orale con colecalciferolo nel migliorare il profilo di rischio cardio-metabolico (sensibilità insulinica, assetto lipidico, markers di infiammazione sistemica, tolleranza glucidica, distribuzione del grasso corporeo)in pazienti con diabete mellito di tipo 2 e diagnosi di NAFLD o NASH.

E.2.3 Trial contains a sub-study: No

E.3 Principal inclusion criteria: Aged between 25 and 65 years

Diagnosis of Type 2 Diabetes Mellitus

Presence of ultrasound detected fatty liver confirmed by MRI

acceptance of informed consent

E.3 Principal inclusion criteria (it): Età compresa tra 25 e 65 anni

Diagnosi di Diabete Mellito di tipo 2

Presenza di steatosi epatica rilevata alla ecografia epatica e confermata dall'esecuzione di RMN

Accettazione consapevole del consenso informato

E.4 Principal exclusion criteria: e <25 or> 65 years

History of alcohol abuse (> 40 mg / day for men,> 20 mg / day for women)

Acute or chronic liver infections autoimmune hepatitis

History of cancer

Other causes of liver disease (hemochromatosis, Wilson's disease)

renal failure

Hyper / hypoparathyroidism

chronic enteropathies

Hypersensitivity to any of the excipients or cholecalciferol hypercalcemia, hypercalciuria (nephrolithiasis, nephrocalcinosis) Therapy with vitamin D, calcium or other drugs affecting bone metabolism, calcium and vitamin D metabolism (anticonvulsants, glucocorticoids, antacids containing aluminum, cholestyramine) Pregnancy and lactation

E.4 Principal exclusion criteria (it): Età < 25 anni o > 65 anni

Storia di abuso alcolico (>40 mg/die per l’uomo, >20 mg/die per la donna)

Infezioni epatiche croniche o acute in atto

Epatite autoimmune

Storia di neoplasie

Altre cause di epatopatie (Emocromatosi, Morbo di Wilson)

Insufficienza renale

Iper/ipoparatiroidismo

Enteropatie croniche

Ipersensibilità al colecalciferolo o a uno qualsiasi degli eccipienti Ipercalcemia, ipercalciuria Calcolosi renale (nefrolitiasi, nefrocalcinosi) Terapia con vitamina D, calcio o altri farmaci che influenzino il metabolismo osteo-calcico e il metabolismo della vitamina D (anticonvulsivanti, glucocorticoidi, antiacidi contenenti alluminio, colestiramina) Gravidanza e allattamento

E.5 End points

E.5.1 Primary end point(s): Reduction of hepatic steatosis in patients with NAFLD and reduction in steatosis, inflammation and liver fibrosis in patients with NASH treated with Vitamin D oral supplementation

E.5.1 Primary end point(s) (it): Riduzione della steatosi epatica nei soggetti affetti da NAFLD e riduzione della steatosi, dell'infiammazione e della fibrosi epatica nei soggetti affetti da NASH dopo trattamento con supplementazione orale di vitamina D

E.5.1.1 Timepoint(s) of evaluation of this end point: 12 months

E.5.1.1 Timepoint(s) of evaluation of this end point (it): 12 mesi

E.5.2 Secondary end point(s): The secondary objective of this study is to assess changes in the cardio-metabolic risk profile in diabetic patients treated with Vitamin D oral supplementation

E.5.2 Secondary end point(s) (it): L'obiettivo secondario dello studio è valutare le modificazioni del profilo di rischio cardio-metabolico

E.5.2.1 Timepoint(s) of evaluation of this end point: 12 months

E.5.2.1 Timepoint(s) of evaluation of this end point (it): 12 mesi

E.6 and E.7 Scope of the trial

E.6 Scope of the Trial

E.6.1 Diagnosis: No

E.6.2 Prophylaxis: No

E.6.3 Therapy: Yes

E.6.4 Safety: No

E.6.5 Efficacy: Yes

E.6.6 Pharmacokinetic: No

E.6.7 Pharmacodynamic: No

E.6.8 Bioequivalence: No

E.6.9 Dose response: No

E.6.10 Pharmacogenetic: No

E.6.11 Pharmacogenomic: No

E.6.12 Pharmacoeconomic: No

E.6.13 Others: No

E.7 Trial type and phase

E.7.1 Human pharmacology (Phase I): No

E.7.1.1 First administration to humans: No

E.7.1.2 Bioequivalence study: No

E.7.1.3 Other: No

E.7.1.3.1 Other trial type description:

E.7.2 Therapeutic exploratory (Phase II): No

E.7.3 Therapeutic confirmatory (Phase III): No

E.7.4 Therapeutic use (Phase IV): Yes

E.8 Design of the trial

E.8.1 Controlled: Yes

E.8.1.1 Randomised: Yes

E.8.1.2 Open: No

E.8.1.3 Single blind: Yes

E.8.1.4 Double blind: Yes

E.8.1.5 Parallel group: Yes

E.8.1.6 Cross over: No

E.8.1.7 Other: No

E.8.2 Comparator of controlled trial

E.8.2.1 Other medicinal product(s): No

E.8.2.2 Placebo: Yes

E.8.2.3 Other: No

E.8.2.4 Number of treatment arms in the trial: 2

E.8.3 The trial involves single site in the Member State concerned: Yes

E.8.4 The trial involves multiple sites in the Member State concerned: No

E.8.4.1 Number of sites anticipated in Member State concerned: 1

E.8.5 The trial involves multiple Member States: No

E.8.6 Trial involving sites outside the EEA

E.8.6.1 Trial being conducted both within and outside the EEA: No

E.8.6.2 Trial being conducted completely outside of the EEA: No

E.8.7 Trial has a data monitoring committee: No

E.8.8 Definition of the end of the trial and justification where it is not the last visit of the last subject undergoing the trial: LVLS

E.8.8 Definition of the end of the trial and justification where it is not the last visit of the last subject undergoing the trial (it): LVLS

E.8.9 Initial estimate of the duration of the trial

E.8.9.1 In the Member State concerned years: 0

E.8.9.1 In the Member State concerned months: 18

E.8.9.1 In the Member State concerned days: 0

F. Population of Trial Subjects

F.1 Age Range

F.1.1 Trial has subjects under 18: No

F.1.1 Number of subjects for this age range: 0

F.1.1.1 In Utero: No

F.1.1.2 Preterm newborn infants (up to gestational age < 37 weeks): No

F.1.1.3 Newborns (0-27 days): No

F.1.1.4 Infants and toddlers (28 days-23 months): No

F.1.1.5 Children (2-11years): No

F.1.1.6 Adolescents (12-17 years): No

F.1.2 Adults (18-64 years): Yes

F.1.2.1 Number of subjects for this age range: 100

F.1.3 Elderly (>=65 years): No

F.2 Gender

F.2.1 Female: Yes

F.2.2 Male: Yes

F.3 Group of trial subjects

F.3.1 Healthy volunteers: No

F.3.2 Patients: Yes

F.3.3 Specific vulnerable populations: No

F.3.3.1 Women of childbearing potential not using contraception : No

F.3.3.2 Women of child-bearing potential using contraception: No

F.3.3.3 Pregnant women: No

F.3.3.4 Nursing women: No

F.3.3.5 Emergency situation: No

F.3.3.6 Subjects incapable of giving consent personally: No

F.3.3.7 Others: No

F.4 Planned number of subjects to be included

F.4.1 In the member state: 100

F.4.2 For a multinational trial

F.5 Plans for treatment or care after the subject has ended the participation in the trial (if it is different from the expected normal treatment of that condition): ORAL VITAMIN D SUPPLEMENTATION WILL BE PERFORMED FOR 6 MONTHS, FOLLOWED BY A 6 MONTHS LASTING FOLLOW UP (MRI EVALUATION OF LIVER STEATOSIS, BIOCHEMICAL MEASUREMENTS OF CARDIO-METABOLIC RISK MARKERS)

F.5 Plans for treatment or care after the subject has ended the participation in the trial (if it is different from the expected normal treatment of that condition) (it): IL TRATTAMENTO CON VITAMINA D AVRA' UNA DURATA DI 6 MESI, SEGUITI DA 6 MESI DI FOLLOW- UP CON VISITE TRIMESTRALI CON VALUTAZIONE DELLE MODIFICAZIONI A CARICO DEL PARENCHIMA EPATICO E DEL PROGFILO DI RISCHIO CARDIO-METABOLICO, COME DESCRITTO NEL PROTOCOLLO

G. Investigator Networks to be involved in the Trial

N. Review by the Competent Authority or Ethics Committee in the country concerned

N. Competent Authority Decision: Authorised

N. Date of Competent Authority Decision: 2011-07-07

N. Ethics Committee Opinion of the trial application: Favourable

N. Ethics Committee Opinion: Reason(s) for unfavourable opinion:

N. Date of Ethics Committee Opinion: 2011-07-07
